# Acute Plasma Biomarkers of T Cell Activation Set-Point Levels and of Disease Progression in HIV-1 Infection

**DOI:** 10.1371/journal.pone.0046143

**Published:** 2012-10-02

**Authors:** Anne-Sophie Liovat, Marie-Anne Rey-Cuillé, Camille Lécuroux, Béatrice Jacquelin, Isabelle Girault, Gaël Petitjean, Yasmine Zitoun, Alain Venet, Françoise Barré-Sinoussi, Pierre Lebon, Laurence Meyer, Martine Sinet, Michaela Müller-Trutwin

**Affiliations:** 1 Institut Pasteur, Unité de Régulation des Infections Rétrovirales, Paris, France; 2 Université Paris Diderot, Paris, France; 3 Institut Pasteur, Unité de Recherche et d'Expertise Epidémiologie des Maladies Emergentes, Paris, France; 4 Institut National de la Santé et de la Recherche Médicale (INSERM) U1012, Régulation de la réponse immune: infection VIH-1 et auto-immunité, Université Paris-Sud, Le Kremlin Bicêtre, France; 5 INSERM U1018, Service d’Epidémiologie et de Santé Publique, AP-HP, Université Paris-Sud, Le Kremlin-Bicêtre, France; 6 AP-HP, Laboratoire de Virologie, CHU Necker-Enfants Malades, Paris, France; 7 Hôpital Cochin-Saint-Vincent de Paul & Université Paris Descartes, Laboratoire de Virologie, Paris, France; University of Cape Town, South Africa

## Abstract

T cell activation levels, viral load and CD4^+^ T cell counts at early stages of HIV-1 infection are predictive of the rate of progression towards AIDS. We evaluated whether the inflammatory profile during primary HIV-1 infection is predictive of the virological and immunological set-points and of disease progression. We quantified 28 plasma proteins during acute and post-acute HIV-1 infection in individuals with known disease progression profiles. Forty-six untreated patients, enrolled during primary HIV-1 infection, were categorized into rapid progressors, progressors and slow progressors according to their spontaneous progression profile over 42 months of follow-up. Already during primary infection, rapid progressors showed a higher number of increased plasma proteins than progressors or slow progressors. The plasma levels of TGF-β1 and IL-18 in primary HIV-1 infection were both positively associated with T cell activation level at set-point (6 months after acute infection) and together able to predict 74% of the T cell activation variation at set-point. Plasma IP-10 was positively and negatively associated with, respectively, T cell activation and CD4^+^ T cell counts at set-point and capable to predict 30% of the CD4^+^ T cell count variation at set-point. Moreover, plasma IP-10 levels during primary infection were predictive of rapid progression. In primary infection, IP-10 was an even better predictor of rapid disease progression than viremia or CD4^+^ T cell levels at this time point. The superior predictive capacity of IP-10 was confirmed in an independent group of 88 HIV-1 infected individuals. Altogether, this study shows that the inflammatory profile in primary HIV-1 infection is associated with T cell activation levels and CD4^+^ T cell counts at set-point. Plasma IP-10 levels were of strong predictive value for rapid disease progression. The data suggest IP-10 being an earlier marker of disease progression than CD4^+^ T cell counts or viremia levels.

## Introduction

Several months after HIV-1 infection, blood CD4^+^ T cell counts, plasma RNA viral load (VL) and CD8^+^ T cell activation levels reach the so-called set-point. At this set-point, which is generally reached after six months post-infection, the levels of the virological and immunological markers, in particular T cell activation levels, are predictive of the rapidity of disease progression [1], [2], [3], [4], [5]. Initiation of antiretroviral treatment is recommended based on CD4^+^ T cell numbers in blood. However, during primary HIV infection (PHI), CD4^+^ T cells decline in blood and VL is generally high. It is therefore difficult to distinguish at this early stage of infection if the low CD4^+^ T cell counts, or the high VL levels, are due to a particularly early diagnosis (close to the VL peak) or rather should be considered as a sign of bad prognosis. New early markers for disease progression could help to determine if an early initiation of treatment is desirable in those patients who are diagnosed during PHI. Moreover, further insight into the early events during HIV-1 infection can help to better understand the mechanisms leading to systemic T cell activation and progressive loss of blood CD4^+^ T cells.

HIV-1 infection is characterized by a cytokine storm in the plasma in the first weeks following infection [6]. The study of the events occurring before and around the VL peak is possible in humans, but only occasionally [6], [7], [8]. As an alternative, the study of SIV infections in non-human primates has allowed the field to gain crucial knowledge of the events occurring early in infection. Pathogenic SIVmac infection in macaques is characterized by a cytokine storm similar to the one described in HIV-1 infection [9]. In contrast, during non-pathogenic infection in natural hosts of SIV, not as many cytokines are induced, and for those that are induced, this induction is transient [10], [11], [12], [13], [14]. Such a transiently increased cytokine is IFN-α [12], [15], [16], [17]. Concomitantly with IFN-α, both macaques and natural hosts show a strong induction of IFN-stimulated genes (ISG) like *IP-10* (*CXCL10*) during primary infection [15], [18], [19]. Again, these ISG inductions are persistent in macaques, as in HIV-infected humans, but only transient in natural hosts. Indeed, in the latter their expressions are rapidly down-regulated [15], [16], [17], [19]. By four weeks post-infection, only macaques, but not natural hosts, display significantly increased plasma cytokines and ISG expressions.

It has been proposed that a particularly early induction of anti-inflammatory proteins could help to down-regulate inflammation, while their chronic production most likely contributes to immunodeficiency [20]. In line with this, in natural hosts, anti-inflammatory factors (IL-10, TGF-β1, PD-1), when induced, are detected very early but do not persist [10], [11], [15], [21].

The data on the differences in primary infection between pathogenic and non-pathogenic SIV infection has led us to raise the hypothesis that a strong inflammation, an uncontrolled IFN response and/or a lack of early anti-inflammatory factors, could be of bad prognosis for disease progression. Previous studies in humans have largely analyzed the association between inflammation and disease progression in the chronic phase of HIV infection. More recently, inflammatory profiles have also been studied more extensively in primary HIV-1 infection [6], [7], [22], [23]. However, only two studies addressed the role of acute inflammation on disease progression markers [24], [25]. None of the studies included a long term follow up of the patients and no data are available on the predictive value of inflammation in PHI for T cell activation levels.

Here, we aimed to analyze whether a specific pro- or anti-inflammatory profile during PHI is predictive of T cell activation and disease progression in patients followed for several years without treatment, in a well-characterized cohort of patients enrolled in PHI [4], [26]. The early levels of 28 plasma proteins, including IFN-I inducible proteins and anti-inflammatory cytokines, were quantified in patients for which disease progression profiles were known. We analyzed whether there was a correlation between these pro- and anti-inflammatory proteins in PHI and disease progression markers, including T cell activation, at set-point. We also evaluated whether the concentrations of these proteins could predict the disease progression profile. Altogether, our study reveals for the first time an association between inflammation in PHI and T cell activation at set-point. Furthermore, IP-10 was an independent predictor of rapid disease progression and our data suggest that, during PHI, IP-10′s capacity of prediction is stronger than that of VL and CD4^+^ T cell counts.

## Results

### HIV-1 Infected Individuals Enrolled in PHI and their Disease Progression Characteristics

**Figure 1:**
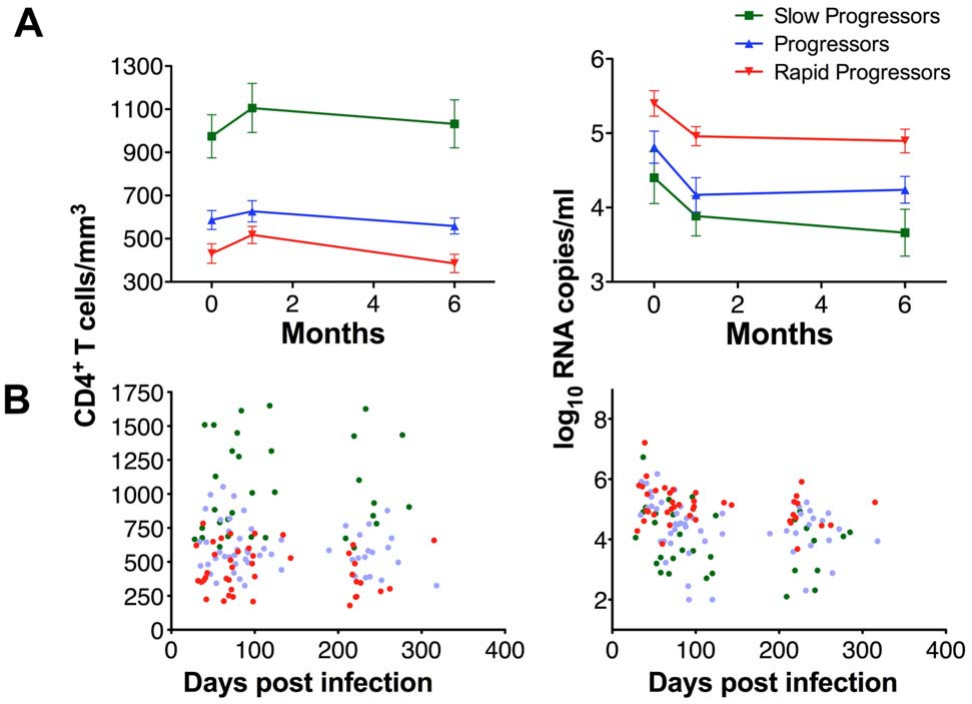
Virological and immunological characteristics of the 46 HIV-1 infected patients with three distinct disease progression profiles. (**A**) CD4^+^ T cell counts (left) and viremia levels (right) are shown over time for the 46 patients, who were divided into 3 groups: slow progressors (in green, 11 patients), progressors (in blue, 19 patients), rapid progressors (in red, 16 patients). The data were determined in primary infection (M0), as well as 1 month (M1) and 6 months (M6) later. The data of the patients are grouped together and the mean and SEM values are shown for each analyzed time point (M0, M1, M6). (**B**) The CD4^+^ T cell counts (left) and viremia levels (right) are shown for each single patient according to the estimated time post-infection.

In order to search whether there is an association between early inflammatory profiles and disease progression, we quantified a wide range of proteins (N = 28) in the plasma of a cohort of 46 patients (Table S1). The patients studied were part of the large, very well characterized ANRS PRIMO cohort [4], [26], [27]. In this cohort, all subjects were enrolled at the stage of PHI. PHI and time post-infection was defined as previously described [4] and as explained in the Material and Methods section below. The majority of the patients were enrolled at Fiebig stage III-IV, which is the most common situation in clinics regarding PHI. We retrospectively selected among antiretroviral treatment-naive patients those who displayed clearly distinct disease progression profiles based on their CD4^+^ T cell counts and/or viremia levels (Figure 1, Table S1). The patients were categorized into three groups: 16 rapid progressors (RP), 19 progressors (P) and 11 slow progressors (SP). The groups were defined based on their CD4^+^ T cell decline over the years during the follow up (42 months post-PHI), as described in the Material section. The three patient groups were similar regarding age, gender distribution and estimated time since infection, while CD4^+^ T cell counts were different at M0 and at set-point (M6) between all groups, and viremia levels differed significantly between RP and SP at all time points (Table S1, Table S2).

Plasma samples were studied at three time points: during PHI (M0) and 1 month later (M1), in order to consider two early time points, as well as six months later (M6), in order to dispose of data at a time, which is generally considered to correspond to the set-point. Plasma samples were available for all 46 patients at M0 and M1, and for 40 out of the 46 patients at M6 (12 RP, 18 P and 10 SP). All plasma samples analyzed were from antiretroviral treatment-naive patients.

### Eight Plasma Proteins were Increased during Primary HIV-1 Infection

**Figure 2:**
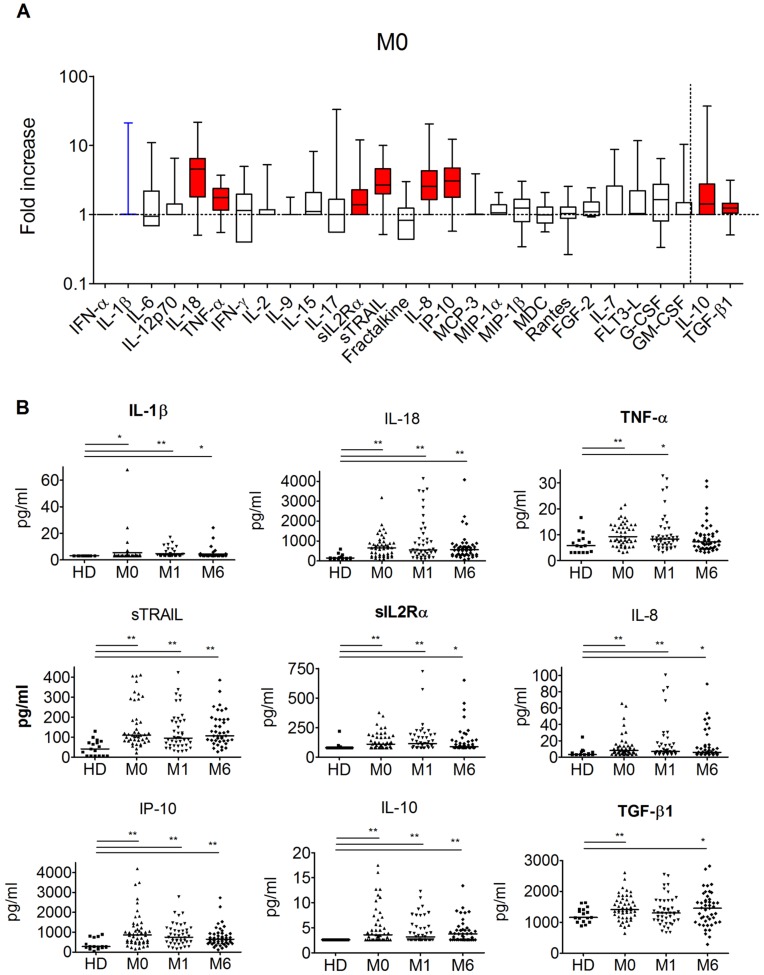
Plasma protein levels in HIV-1 infected patients. (**A**) The levels of 28 proteins in the plasma of 46 acutely infected patients (M0) are expressed as fold change compared to the levels in healthy donors (N = 17). The order of the proteins is presented according to their function (pro-inflammatory, adaptive, IFN-inducible, chemoattractants, hematopoietic and anti-inflammatory). The anti-inflammatory cytokines are presented on the right side of the figure. The dotted horizontal line at Y = 1 corresponds to the value in healthy donors. The boxes represent the median and the 25^th^ and 75^th^ percentile, with the line in the middle of the boxes corresponding to the median value. Colored boxes stand for the cytokines, whose levels were significantly different from healthy donors: blue boxes when p<0.05 (as it was the case for IL-1β) and red boxes when p<0.008 (M&W test). (**B**) Protein concentrations at M0, M1 and M6 of the soluble factors that were elevated at M0. The data are expressed in pg/ml, HD: healthy donors, M: months. Cytokine concentrations below the limit of detection were arbitrarily set at the level of the limit of detection. Dot-plots marked with one asterisk (*) (p<0.05) or two asteriks (**) (p<0.008) represent the cytokines, whose levels were significantly different from healthy donors (M&W test).

We quantified the concentrations of each of the 28 proteins in the 46 HIV-1 infected individuals and evaluated whether their levels were different or not as compared to the respective protein levels in healthy donors. The proteins examined were chosen based on following criteria: they are either known to have a pro- or anti-inflammatory role, to be inducible by IFN, to be increased during PHI or to be associated with disease progression during chronic HIV-1 and SIVmac primary infections [6], [9], [10], [11], [15]. At M0, 8 soluble factors were significantly more elevated than in non-infected individuals: IL-18, TNF-α, sIL2Rα, sTRAIL, IL-8, IP-10, IL-10 and TGF-β1 (FDR, p<0.008) (Figure 2A). All proteins that were increased at M0 were also increased at M1 and M6 with respect to healthy donors, with the exceptions of TGF-β1 at M1 and TNF-α at M6 (Figure 2B). No other plasma proteins were increased at M1 and M6 besides those already increased at M0. All plasma samples were negative for IFN-α except for one rapid progressor at M6 (data not shown).

Altogether, 6 pro- and 2 anti-inflammatory soluble factors out of the 28 tested were increased during PHI.

### Rapid Progressors Presented the Highest Number of Elevated Pro- and Anti-inflammatory Proteins during Primary HIV-1 Infection

**Figure 3:**
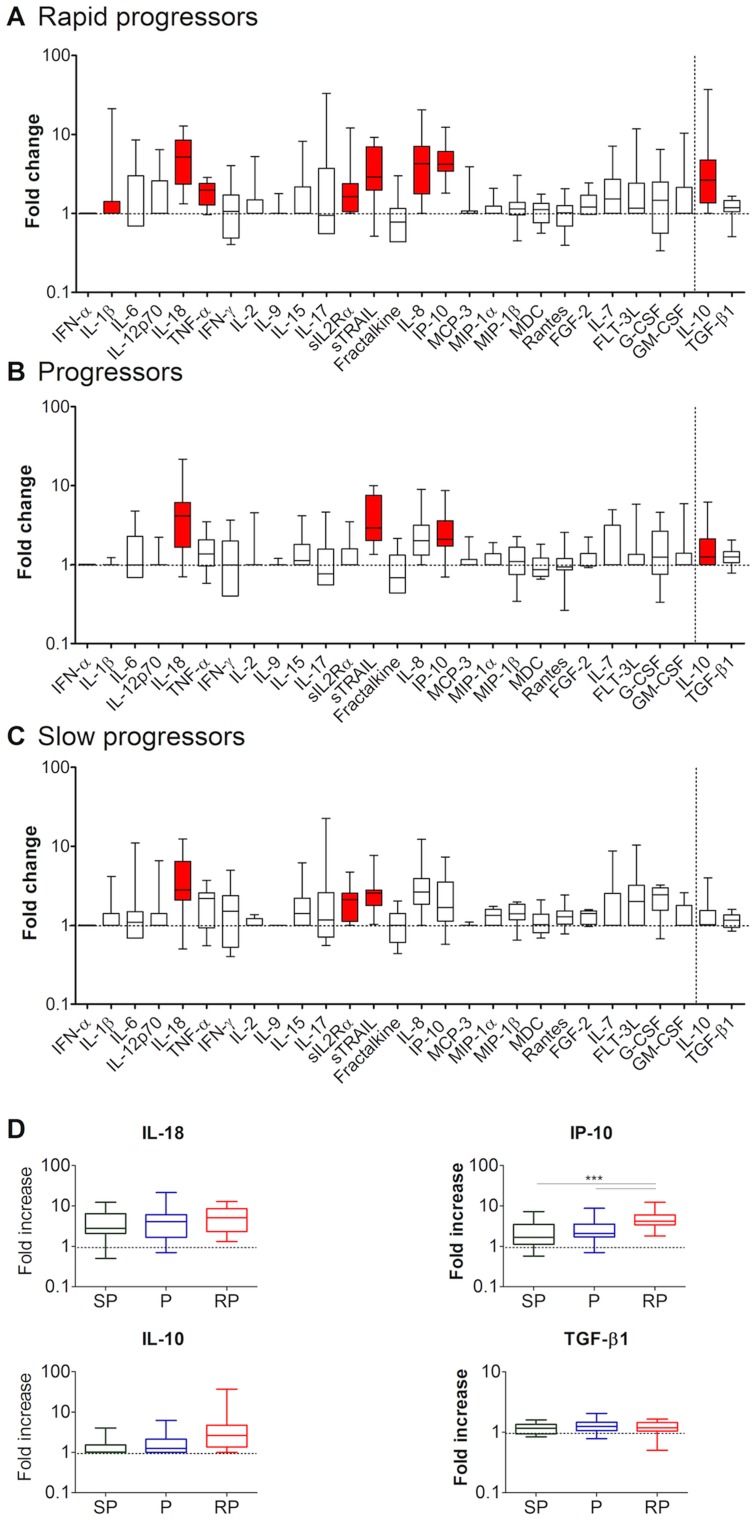
Plasma protein levels at M0 according to disease progression profiles. The cytokine profiles at M0 are shown for each group of patients: 16 rapid progressors (**A**), 19 progressors (**B**), and 11 slow progressors (**C**). Color code and statistical analyses are as described in Figure 2 (corrected threshold, p<0.005). The dotted horizontal line corresponds to the value in healthy donors. (**D**) Comparison of protein concentrations between the 3 groups of patients (SP, P and RP). Four representative cytokines are shown. The cytokines increasing significantly over groups were IP-10 and IL-10 (Cuzick’s test, p<0.007). When comparing the groups two by two, out of 28 proteins tested, the levels were different only for IP-10 (M&W test,***: p<0.005).

We then investigated whether there were differences in the protein concentrations according to the disease progression profile. We compared the concentrations of the 28 soluble factors in each group of patients, i.e. rapid progressors (RP), progressors (P) and slow progressors (SP) to those in healthy donors. Interesting differences according to the disease progression profile of the patients were observed. Thus, in RP, 8 proteins were significantly elevated at M0 (Figure 3A and Table S3). These analytes were the same proteins that were increased when all patients were grouped together (Figure 2), except for TGF-β1, which was not elevated in any of the patient groups when comparing them to healthy donors separately. In contrast to RP, P and SP displayed elevations for only 4 and 3 proteins, respectively (Figure 3B + C and Table S3) (FDR, p<0.005). At M1 and M6, there were again more soluble factors elevated in RP (N = 4) than in P (N = 2 at M1 and M6) and SP (N = 1 at M1 and N = 3 at M6) (FDR, p<0.002) (Table S3 & Figure S1 *).* Cytokines which were increased in RP or P, but not in SP were: TNF-α, IL-1β, IL-8, IP-10 and IL-10.

In conclusion, starting from M0, RP had a higher number of elevated soluble factors than the other two patient groups (P and SP).

### Plasma IP-10 Concentrations in Primary Infection were Different between Groups of Patients Progressing at Distinct Rates

We then addressed the question of whether the concentrations of these proteins differed between the patient groups. We first analyzed the differences in protein concentrations by comparing all three groups concurrently. The analysis was performed using the Cuzick’s test, which allows multiple, ordered group comparisons. Two out of the 28 proteins showed increases over the groups (from the lowest levels in the SP, followed by the P and highest in the RP) at all time points (M0, M1, M6). These were IP-10 (p<0.007) and IL-10 (p<0.001) (Figure 3D for M0, and data not shown for M1 and M6).

We further explored the differences by comparing the groups two-by-two. Only for IP-10 there was a significant difference in the cytokine concentration between two groups of patients (Figure 3D). The concentrations of IP-10 were indeed different between RP and P, and also between RP and SP (p<0.005). These differences were observed at all time points (M0, M1, M6) (Figure 3D for M0, and data not shown for M1 and M6).

### IP-10, TGF-β1 and IL-18 in PHI were Associated with T Cell Activation at Set-point

**Figure 4:**
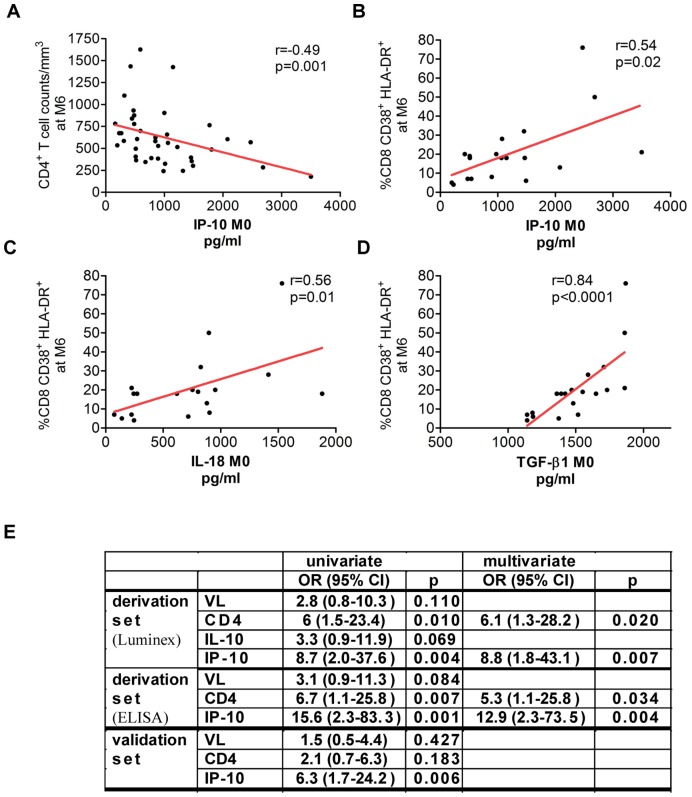
Cytokines predictive of immunological set-point levels. (A–E) Cytokine concentrations in plasma at M0 have been plotted against CD4^+^ T cell counts and T cell activation (CD3^+^CD8^+^CD38^+^HLA-DR^+^) at M6. Six patients, including 4 who were treated at M6, were excluded from the analysis at M6. T cell activation levels were available for 19 patients at M6 (4 SP, 7 SP, 8 RP). The correlations were thus analyzed in 40 patients regarding CD4^+^ T cell counts and viral load and for 19 patients regarding T cell activation. (**A**) IP-10 levels at M0 plotted against T CD4^+^ counts at M6. (**B**) IP-10 levels at M0 plotted against T cell activation at M6. (**C**) IL-18 levels at M0 plotted against T cell activation at M6. (**D)** TGF-β1 concentrations at M0 plotted against T cell activation at M6. The red line indicates that both the Spearman correlation and the linear regression analysis were significant. **(E)** Regression analysis for evaluation of the capacity of CD4^+^ T cell counts, VL and cytokines at M0 to predict rapid disease progression. Values obtained for both the derivation set (Luminex and ELISA) and the validation set are shown. Median values of CD4^+^ T cell counts, VL and cytokine levels were used: VL > = 5 log; CD4<570 cells (derivation set); CD4<546 cells (validation set); IP-10> = 869 pg/ml (derivation set, Luminex); IP-10> = 247 pg/ml (derivation set, ELISA); IP-10> = 232 pg/ml (validation set, ELISA).

To analyze if the cytokine levels in PHI were associated with markers of disease progression, we evaluated whether the concentrations of one or more of the 28 proteins at M0 correlate with viremia, CD4^+^ T cell counts and/or T cell activation levels at set-point (M6). Three cytokines (IP-10, IL-18 and TGF-β1) measured at M0 correlated positively with T cell activation at M6 (Figure 4 B, C and D). Two cytokines (IP-10 and IL-10) evaluated at M0 were negatively correlated with CD4^+^ T cell counts at M6 (Figure 4A and Figure S2). IL-10 levels at M0 were associated with VL at M6 (Figure S2).

Among all comparisons examined, the best correlations (rho≥0.5) were observed for IP-10, IL-18 and TGF-β1 (at M0) with T cell activation (at M6).

### IP-10 and TGF-β1 were Predictive Factors for Respectively CD4^+^ T Cell Counts and T Cell Activation at Set-point

To analyze whether some of the inflammatory cytokines were not only associated, but also predictive for one or more of the three disease progression markers, we performed multivariate linear regression analyses. After multiple adjustments, none of the 28 proteins in PHI was identified as a predictor for VL at M6. TGFβ-1 and IL-18 at M0 were both positively associated with T cell activation at M6 (p<0.001 and p = 0.020, respectively). Their concentration at M0 predicted as much as 74% (adjusted R^2^ = 0.74) of the T cell activation values at M6, 66% being predicted by TGF-β1 alone. IP-10 levels at M0 were negatively associated with CD4^+^ T cell counts at M6 (p = 0.021). IP-10 concentration at M0 predicted 30% (adjusted R^2^ = 0.3) of the CD4^+^ T cell count variation at M6.

### Plasma IP-10 Levels in Primary Infection Predicted Rapid Disease Progression

Next, we studied if the concentrations of one or more of the pro- and anti-inflammatory proteins, increased in PHI, were predictive of disease progression. Additionally, we aimed to compare the capacity of prediction of the soluble proteins with that of CD4^+^ T cell counts and VL at M0. Univariate logistic regressions were performed to analyze if the plasma proteins, CD4^+^ T cell counts and VL, categorized according to their respective median values at M0, were predictive either for slow progression (SP versus P and RP) or for rapid progression (RP versus P and SP). In the univariate logistic regression analysis, plasma IP-10 concentration (≥869 pg/ml), CD4^+^ T cell counts (<570 cells/mm^3^), VL (≥5 log_10_ RNA copies/ml) and plasma IL-10 (≥3.7 pg/ml) at M0 were identified as potentially predictive of rapid disease progression (Figure 4E). This was not the case neither for IL-18 levels (≥673 pg/ml) nor for TGF-β1 levels (≥1533 pg/ml). The logistic regression model revealed that only IP-10 (p = 0.007) and CD4^+^ T cell counts (p = 0.020) were independent predictors for rapid disease progression (Figure 4E).

### Validation of the Results Obtained by Luminex on IP-10 by ELISA

It was not anticipated that early plasma IP-10 levels could be of better predictive value than VL and perhaps also slightly better than CD4^+^ T cell counts in PHI. In order to confirm these results, we validated the data obtained with the Luminex technology by using another, more classical technique for quantification of proteins, i.e. an Elisa assay. The IP-10 plasma concentration at M0 was quantified by ELISA in 45 out of the 46 patients (Table 1). For one patient, no plasma was available for the ELISA. The prediction values for IP-10 didn’t change using these 45 patients (*not shown*). The IP-10 concentration, as determined by ELISA (median of 249 pg/ml, range: 2–318), strongly correlated with those measured by Luminex (r = 0.79, p<0.001) (Figure S3).

**Table 1:** Comparative distribution of patient’s demographic, clinical and immunological characteristics in the derivation and validation sets.

	Derivation set, N = 45	Validation set, N = 88	p
**Gender W/M (% of women)**	9/45 (20%)	9/88 (10%)	0.12
**Age at enrollment (years)**	35 (18–64)	36 (18–67)	0.71
**Estimated time post-infection at M0 (days)**	51 (28–98)	55 (28–98)	0.83
**Blood CD4^+^ T cells/mm^3^ (range)**	571 (208–1509)	546 (250–1295)	0.94
**Plasma viral load (log copies of RNA/ml)**	5.03 (2.45–7.21)	4.9 (2.41–7.41)	0.63
**IP-10 (pg/ml)**	249 (2–1318)	232 (40–2880)	0.79

The main characteristics of the patients are listed here for the time point of enrollment during PHI (M0). For each parameter (except gender), the median values and the range are indicated. Plasma IP-10 concentrations were measured by ELISA at M0. For the derivation set, data are shown for those 45 patients out of 46, whose IP-10 was quantified by ELISA. The validation set comprised 88 patients. There was no significant difference between the two sets for any parameter (p>0.11). W: women, M: men, N: number of patients in each set.

Univariate regression analyses were then performed. CD4^+^ T cell counts <570 cells/µl (p = 0.007) and in particular IP-10≥249 pg/ml (p = 0.001) were associated with rapid disease progression, while VL ≥5 log_10_ RNA copies/ml only showed a trend for significance (p = 0.084) (Figure 4E). In the multivariate regression analysis, IP-10 (p = 0.004) and CD4^+^ T cells (p = 0.034) were independently predictive of rapid progression (Figure 4 E).

Thus, the results based on the ELISA were similar to the ones obtained with the Luminex platform. In both cases, VL in PHI was not significantly predictive for rapid disease progression, while CD4^+^ T cells and to a greater extent IP-10 were.

### Validation of Plasma IP-10 Levels in PHI as a Predictive Factor of Disease Progression

In order to further validate the capacity of early prediction by IP-10, we replicated the analysis in a blind fashion on an independent and larger set of patients (N = 88), called the validation set (Table 1). In contrast to the previous set of patients (derivation set), the patients of the validation set were not selected based on distinct profiles of disease progression, but enrolled in a random manner. They were recruited more recently than patients of the derivation set and followed for at least 12 months. All were antiretroviral treatment-naïve.

Plasma IP-10 concentrations during PHI were measured by ELISA in the 88 HIV-1 infected individuals. The median value of IP-10 in this validation set (232 pg/ml) was not different from the one in the derivation set (249 pg/ml) (p = 0.79, Table 1).

We then classified the patients according to their disease progression profiles, based on their CD4^+^ T cell counts over time, as above. As these patients were recruited more recently than the patients from the derivation set and had been followed for a shorter period of time, we were only able to define who was a RP, but could not differentiate between P and SP. However, since the aim of this analysis was to determine the predictive value of plasma IP-10 in PHI for rapid disease progression, this did not constitute an obstacle for our study. The 88 patients split into 17 RP and 71 P/SP. As one could have expected, the proportion of RP in this validation set was lower (19.3%) than in the previous set (34.8%) (p = 0.04). For the previous one, we had selected on purpose similar numbers of individuals in each category of disease progression. In the second set, individuals were enrolled blindly without knowing their disease progression profile. The proportion of RP within the validation set mirrored the normal frequency of RP in HIV-1 infected individuals [4], [28].

**Figure 5:**
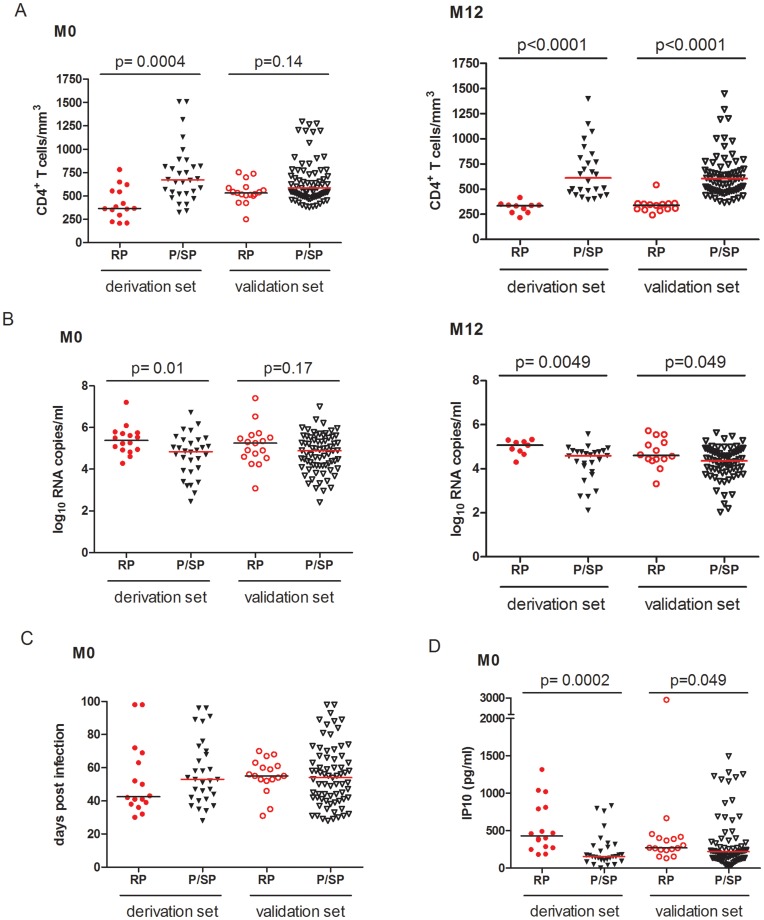
Immunological, virological and clinical characteristics of rapid progressors versus progressors and slow progressors assembled within patients of the derivation and validation sets. The derivation set comprised 46 patients (except for IP-10, where values based on the ELISA were available for only 45 patients). The validation set corresponded to 88 patients for all markers. Differences between rapid progressors and the other patients are indicated with the presence of a p value (M&W test). P values below 0.05 were considered to be significant. There was no significant difference regarding the time of enrollment at M0 (estimated days post-infection). (A) CD4^+^ T cells at M0 and M12; (B) Viremia at M0 and M12; (C) number of estimated days post-infection at M0; (D) Plasma IP-10 concentration at M0. M = month.

There were no significant differences between the derivation and validation sets in regard to age, gender, estimated time since infection, CD4^+^ T cell counts or viremia at M0 (p>0.11) (Table 1). In the validation set, the CD4^+^ T cell counts and viremia levels at M0 were not different when comparing RP to P/SP (P and SP assembled), in contrast to the derivation set, where CD4^+^ T cells, as well as viremia levels, were different between RP and P/SP already at M0 (Figure 5). This is in line with the random enrollment of the patients from the validation set. Of note, plasma IP-10 levels were higher in RP versus P/SP at M0 in the validation set (p = 0.049), in contrast to CD4^+^ T cell counts and viremia (Figure 5 D). The difference in IP-10 levels was though less pronounced than in the derivation set (p = 0.0002).

Univariate regression analyses were then performed on this independent set of patients, in order to evaluate the capacity of plasma IP-10, CD4^+^ T cell counts and viremia to predict rapid disease progression. The analysis was again based on the median IP-10 concentration (≥232 pg/ml). Only IP-10 was significant (p = 0.006), while CD4^+^ T cell counts and viremia at M0 were not (Figure 4 E). This was also observed with another categorization of IP-10, i.e. 3 categories of IP-10 based on the median and third quartile *(not shown).* This confirms IP-10 as being a strong predictive factor of rapid disease progression at this early stage of infection (PHI).

Overall, the analysis in this independent group of patients demonstrates that elevated plasma IP-10 level during PHI is of robust predictive value for rapid evolution towards AIDS. They confirm the data described above, which already suggested that during PHI, IP-10 is of stronger prognosis for disease progression than viremia levels or CD4^+^ T cell counts.

## Discussion

During chronic HIV-1 infection, many plasma proteins and activation markers, such as neopterin, TNF-α and sCD14, are associated with progression towards AIDS [9], [29]. Here we studied which plasma proteins during PHI are predictive of the virological and immunological set-points and of disease progression itself. As published by others, we detected an increase in many proteins (such as IP-10, IL-8, IL-10, IL-18, TNF-α, sIL2Rα) during PHI. These included the same as those previously reported [6], [7], [23], [24], [30]. We did not detect increases of a few other proteins, reported in some studies [6], [7]. For instance, we did not observe increased levels of two cytokines, IFN-α and IL-15, known to be produced very early on in PHI. This could be explained by the fact that the time points analyzed here corresponded mostly to Fiebig stages III and IV. Of note, we analyzed two soluble factors, TGF-β1 and sTRAIL, that to our best knowledge have not been measured before in the plasma during PHI. We observed increases for both of these proteins, corresponding to data obtained in non-human primate models [10], [11], [31], [32]. Most importantly, this is the first time that acute inflammatory protein levels were examined for their predictive value for T cell activation.

We show here that RP have an increase in both the breadth and concentration of cytokines induced during PHI as compared to the other patients groups, and that RP and P, but not SP, presented early elevation of both pro- (IP-10) and anti-inflammatory (IL-10) cytokines. It could appear counterintuitive to observe that anti-inflammatory cytokines were higher in RP and P than in SP already in PHI, since they should rather dampen the inflammation. However, it has been reported that IL-10 is induced in a later stage of PHI, after the first burst of pro-inflammatory cytokines, both in HIV-1 and SIVmac infections [6], [7], [9]. The anti-inflammatory cytokines may thus have been induced as a consequence of the strong inflammatory response, that is more pronounced in progressors. Chronic TGF-β1 and IL-10 production might then participate in disease progression, for instance by inhibiting adaptive immune responses [20]. TGF-β1 together with IL-18 predicted as much as 74% of the T cell activation at set-point. IL-18 and TGF-β1 have never been studied before for their predictive value at the early stage of HIV-1 infection. Excessive TGF-β1 expression in lymph nodes, through the induction of fibrosis, has been suggested to play a predominant role in the disruption of lymph node architecture and CD4^+^ T cell depletion in HIV and SIV infections [31], [33]. IL-18 is known to induce IFN-γ expression, which in turn is capable of inducing IP-10 [34].

IP-10 is shown here to be an independent predictor of rapid disease progression. This contrasted with the other cytokines, and even with VL and CD4^+^ T cell counts, which at this early stage of infection had, respectively, little or no predictive power. Our study is not in contradiction with earlier data on VL and CD4^+^ T cell counts in PHI as markers of disease progression [4], but suggests that IP-10 is of stronger predictive value than VL and CD4^+^ T cell counts in PHI. The predictive capacities of VL and CD4^+^ T cell counts have been analyzed in many studies with large sample sizes [35]. The role of IP-10 as an early independent predictor of rapid progression was detected here despite the relatively small number of patients, supporting its strength. In addition, the data have been validated in an independent group of patients.

IP-10 has stood out in many other studies. Thus, it has been previously shown that IP-10 strongly correlates with viremia in PHI [24], which was also the case here (p = 0.004, *not shown*). In the chronic phase of HIV-1 infection, IP-10 showed a strong positive correlation with VL and a negative one with CD4^+^ T cell counts [36], [37]. Studies in patients undergoing HAART pinpointed IP-10 to be better correlated with controlled infection than several other inflammatory markers. In a study where patients started treatment during PHI, only IP-10 significantly differed between treated and untreated participants at week 16–24 [7]. Similarly, after Mega-HAART was initiated during PHI, IP-10 was decreased, whereas other markers, i.e. sCD14 and LPS, were not [22].

Only two studies however evaluated the capacity of IP-10 to predict disease progression (over a period of 18 and 24 months), and contrasting results were reported [24], [25]. In the study by Roberts *et al*, the first study that ever evaluated the role of acute inflammation for CD4^+^ T cell loss, IP-10 was not identified as an early predictive marker for CD4^+^ loss [24]. The study was performed in a cohort of women in South Africa, while in the ANRS PRIMO cohort, the percentage of patients from sub-saharan Africa corresponds to only 6.5%, with the vast majority of patients being Caucasian men (Table 1) [26], [27]. Sexually transmitted infections were frequent among the South-African women studied, since 94.5% of them were carriers of at least one infection [24]. Mucosal inflammation can impact on peripheral markers [23]. Further studies are needed to evaluate whether there could be a difference with respect to IP-10 as a biomarker between men and women and in the baseline levels between cohorts in Europe and Africa [36]. More recently, a study in a cohort of HIV-1 infected men in China has found IP-10 as an early predictor of rapid disease progression [25]. This is interesting as it shows that IP-10 has a similar role as a biomarker in a cohort with a distinct geographic environment and distinct genetic background. However, in that study, they did not compare the efficacy of IP-10 as a biomarker of rapid progression to that of CD4^+^ T cell counts and VL. They also did not evaluate the capacity of cytokines to predict T cell activation.

IP-10 is inducible by IFN-II, but also by other factors, such as IFN-I [15], [38]. In SIVmac-infected macaques, a sustained production of IFN-I can be observed in lymph nodes [14], [17], [39], [40]. It is possible that a prolonged production of IFN-I in lymphoid organs during HIV-1 infection is responsible for the heightened levels of IP-10. It also not excluded that IP-10 production subsequent to the early peak of IFN-I is fueled by other factors, such as IFN-γ or TNF-α [41]. IP-10 is the ligand of CXCR3, which is expressed on many cells (activated T cells, Th1 cells, Treg cells, plasmacytoid dendritic cells, monocytes, NK cells) and is implicated in the recruitment of CXCR3^+^ cells to lymph nodes [42], [43], [44]. Events in lymph nodes might have a particular impact on immunodeficiency [45]. IP-10 is known to be produced in secondary lymphoid organs of viremic HIV-1 infected individuals and elevated levels in lymph nodes of chronically infected macaques are associated with more rapid disease progression [15], [18], [42], [46]. Production of IP-10 in lymph nodes might amplify the inflammation and the spread of viral infection by attracting potential target cells of HIV [15], [42]. IP-10 enhances HIV-1 replication in monocyte-derived macrophages and primary CD4^+^ T cells *in vitro* [47]. IP-10 levels have been shown to correlate with the frequency of pro-inflammatory CD16^+^ monocytes [37]. It has been suggested that Treg cells, which accumulate in lymph nodes of progressors, are the major producers of TGF-β1 in lymph nodes [31], [48]. Treg cells can express CXCR3 and these cells could thus be attracted by IP-10. Further studies will be needed to investigate whether there is a link between IP-10 and TGF-β1 in lymph nodes and whether IP-10 is only a strong marker of inflammation and disease progression or whether it also contributes by itself to immunodeficiency.

Once individuals are diagnosed for HIV infection during PHI, one of the most pressing decisions confronted by the clinicians is whether or not to start antiretroviral therapy. Although early treatment is most likely beneficial for limiting the size of viral reservoirs, long-term treatment is a burden for the patient. None of the currently available biomarkers has a perfect sensitivity or specificity. For instance, VL explains <50% of the variation in time from primary infection to the development of clinical AIDS [49]. Novel early biomarkers, in addition or combination with the canonical parameters, could help clinicians to refine the decision. Furthermore, soluble plasma proteins, such as IP-10, would be ideal biomarkers to assess as they are abundant and easily measurable.

In conclusion, we show for the first time that the level of inflammation in primary HIV-1 infection is predictive of T cell activation levels. This underlines the major role of inflammation in driving T cell activation, as previously suggested by others [17],[50]. Our study highlights the role of TGF-β1 and two IFN-inducible cytokines (IP10 and IL-18) in determining the immunological set-points during HIV-1 infection. The plasma concentration of one single cytokine, IP-10, could even predict on its own rapid disease progression. Of note, IP-10 level during PHI was better predictive of rapid progression than viremia or CD4^+^ T cells counts. IP-10 therefore seems to be a particularly strong early prognosis factor. It is unknown so far if and which role IP-10 plays in pathogenesis, but it could be a particular sensitive indicator for the level of harmful immune activation during HIV-1 infection.

## Methods

### Patients

The patients were part of a large, multi-center cohort, named PRIMO cohort CO06, funded by the French governmental agency of AIDS research (ANRS) [4], [26], [27]. The study was approved by the national Ethics Committee (CCPPRB Paris-Cochin, N°1157/18-6-96) and by the Biomedical Research Committee of Institut Pasteur (2007.020). All patients have given their written informed consent.

We first performed a retrospective study on 46 patients (Figure 1 and Table S1). These subjects were enrolled between 1999 and 2006, at the stage of PHI. Briefly, in order to be in PHI, the individuals had to be in the first three months of infection (here between days 28 and 90 p.i.) with either an incomplete Western blot, or with a positive p24 antigenemia or detectable HIV RNA associated with a negative Western blot or a weakly positive ELISA [4], [26], [27]. The time since infection, when unknown, has been estimated as previously described [4], [26].

Retroviral treatment-naive patients, showing distinct contrasting profiles in their spontaneous CD4^+^ T cell count declines and viremia levels during a retrospective follow up of 42 months, were selected for this study (Figure 1 and Table S1). They were divided into three groups based on their CD4^+^ T cell profiles: Rapid progressors (RP) were defined as those loosing their peripheral CD4^+^ T cells to below 350 cells/mm^3^ in less than 12 months (16 patients); Progressors (P) showed more than 350 CD4^+^ T cells/mm^3^ after 12 months but less than 500 cells/mm^3^ at least at one time point before or at 42 months post-PHI, in the absence of treatment (19 patients); and Slow progressors (SP) were displaying more than 500 CD4^+^ T cells/mm^3^ after 42 months post-PHI in the absence of treatment (11 patients). We analyzed plasma samples from blood collected on EDTA at time of diagnosis (M0), as well as 1 month (M1) and 6 months (M6) post-enrollment. None of the patients were treated at M0, and samples from patients treated at M1 or M6 (N = 4) were excluded from the analyses.

A second, independent group of 88 patients was enrolled between 2007 and 2009, at the stage of PHI (Table 1 and Figure 5). Patients were not treated and followed for at least 12 months.

Blood collected on EDTA was also obtained from 17 healthy donors through the French blood bank (EFS) as part of the EFS-Institut Pasteur convention.

### Flow Cytometry

T cell activation was defined as the percentage of activated CD8^+^ T cells (CD3^+^CD8^+^CD38^+^HLA-DR^+^ cells), as described previously [51]. PBMC from 27 and 19 patients (4 SP, 7 P, 8 RP) were available for CD8^+^ T cell activation determination at M0 and M6, respectively.

### Cytokine Quantification

Twenty-four plasma proteins were measured using a human cytokine Milliplex kit (Millipore). For each of the proteins, the limit of detection was considered as the standard point having a median fluorescence intensity two fold higher than the blank (*see Methods S1*). Plasma levels of transforming-growth-factor β1 (TGF-β1, Invitrogen), Interleukin 18 (IL-18, Invitrogen) and soluble TNF-related-apoptosis-inducing-ligand (sTRAIL, R&D) were determined by ELISA, because they were not available on Luminex or the detection limits above the physiological concentrations of the cytokines in the plasma from healthy donors. IP-10 was measured both by Luminex and ELISA (R&D). Titers of bioactive IFN-I were determined with a sensitive, functional assay as previously described [12].

### Statistical Analyses

Global comparisons were performed using the non-parametric Kruskal-Wallis test. Two by two group comparisons were based on the non-parametric Mann and Whitney U-test (M&W) and adjustments for multiple tests were performed using the “False discovery rate” (FDR) [52]. Cuzick's non-parametric test was used to explore trends across multiple groups. Two-by-two correlations were evaluated using Spearman correlation coefficient. Cytokines associated with either VL, CD4^+^ T cell count and/or T cell activation set-point levels in univariate linear regression analyses (p<0.10) were included in the final multivariate models in a stepwise manner to identify independent predictors. For each analysis, the same and thus equal number of patients were used. To satisfy the normality assumption, CD4^+^ T cell counts and T cell activation levels were modeled after log_10_ transformation. Logistic regression analyses were used to estimate the prediction capacity of early cytokine concentrations for CD4^+^ T cell counts and T cell activation levels at set-point, as well as to study the predictivity of cytokine levels for slow progression (SP versus P and RP) or rapid progression (RP versus P and SP). All factors associated in univariate analyses (p<0.10) were included in the multivariate logistic regressions. VL was incorporated into the multivariate models to estimate the prediction for CD4^+^ T cell counts and T cell activation. CD4^+^ T cell numbers were additionally incorporated in the analyses for the prediction of T cell activation levels.

## Supporting Information

Figure S1 **Plasma protein levels at M1 and M6 according to disease progression profiles.** The plasma protein levels at M1 (**A**) and M6 (**B**) were expressed as fold change compared to those in healthy donors. Significant changes are indicated as red boxes (corrected threshold p<0.002). When a cytokine was increased (p<0.05), but the p-value was not under the corrected threshold (p<0.002) it was represented in a yellow box (p<0.005 on **A** and p<0.008 on **B**). The cytokines are listed from left to right according to their role (inflammatory, adaptive, IFN-inducible, chemoattractive, hematopoietic and anti-inflammatory). The dotted horizontal line corresponds to the respective values in healthy donors.
(DOC)

Figure S2 **Correlation between plasma cytokine levels at M0 and disease progression markers.** The cytokine concentrations at M0 have been plotted against (**A**) T CD4^+^ counts (N = 40), (**B**) viremia (N = 40) and (**C**) T cell activation (CD3^+^CD8^+^CD38^+^HLADR^+^, N = 19) levels at set point (M6). Here are represented the 5 cytokines (IP-10, IL-18, MCP-1, IL-10, TGF-β1) correlated with one (or more) of the disease progression markers (Spearman correlation). A red line indicates both a significant correlation and a significant linear regression. A black dotted line represents a non-significant linear regression. VL: viral load.
(DOC)

Figure S3 **Correlation between IP-10 plasma concentrations quantified by Luminex and by Elisa.** The IP-10 concentrations were determined in 45 patients during primary HIV-1 infection.
(DOC)

Table S1 **Demographic, clinical, virological and immunological characteristics of the 46 HIV-1 infected patients belonging to the derivation set.** For each parameter, the median value is indicated. There were no significant differences between the groups concerning age (p = 0.65), gender (p = 0.32) or estimated time since infection (p = 0.4) (M&W test). In contrast, the three groups of patients presented differences in their T CD4^+^ counts and VL levels (see also Table S2). RP: Rapid progressors, P: progressors, SP: Slow progressors. The symbol ^§^ indicates a significant difference between SP and RP, * a difference between P and SP and ^#^ between RP and P (p<0.05). W: women, M: men, N: number of patients in each group.
(DOC)

Table S2 **Comparison of viral load and T CD4^+^ counts between RP, P and SP of the derivation set.** The VL and CD4^+^ T cell counts have been compared between the groups of patients described in Figure 1 and Table S1. The p-values are shown here for each comparison (M&W U-test). In red are indicated the statistically significant differences (p<0.05). M = month, RP = rapid progressor, P = progressor, SP = slow progressor.
(DOC)

Table S3 **Plasma protein profiles according to disease progression.** The plasma proteins significantly elevated in one or more groups (RP for rapid progressors, P for progressors and SP for slow progressors) are shown here for the three time points: M0 (primary infection), M1 and M6. “**+**” stands for a significant difference as compared to healthy donors (M&W test, FDR corrected threshold: p<0.005 at M0, p<0.002 at M1 and M6). A “-“ stands for no change. M = month.
(DOC)

Methods S1 **Luminex assay.** Twenty-four plasma proteins were measured using a human cytokine Milliplex kit (Millipore): Fibroblast-growth-factor 2 (FGF-2), FMS-like-tyrosine-kinase-3-Ligand (FLT-3L), Fractalkine, Granulocyte-colony-stimulating factor (G-CSF), Granulocyte-macrophage-colony-stimulating factor (GM-CSF), Interferon γ (IFN-γ), Interleukin 1 β (IL-1β), IL-2, IL-6, IL-7, IL-8, IL-9, IL-10, IL-12p70, IL-15, IL-17, IP-10, macrophage-derived-chemokine (MDC), macrophage-inflammatory-protein 1α and β (MIP-1α, MIP-1β), monocyte-chemotactic-protein 3 (MCP-3), regulated-upon-activation-normal-T-cell-expressed and secreted (Rantes), soluble-IL2 receptor α (sIL2Rα) and tumor-necrosis-factor α (TNF-α). The limit of detection was 3.2 pg/ml except for: FLT-3L, G-CSF, GM-CSF, IL-7 and MIP-1β (16 pg/ml); FGF-2, MCP-3 and MIP-1α (40 pg/ml); and IP-10, Fractalkine and sIL2Rα (80 pg/ml). The standards (3.2 to 10,000 pg/ml) of the manufacturer were run on each plate in duplicate. All samples were assayed concurrently to avoid inter-assay variability. Data were acquired using a Luminex-100 system and analyzed using Bio-Plex Manager Software (Applied-Cytometry).
(DOC)

## References

[1] Giorgi JV, Hultin LE, McKeating JA, Johnson TD, Owens B, et al (1999) Shorter survival in advanced human immunodeficiency virus type 1 infection is more closely associated with T lymphocyte activation than with plasma virus burden or virus chemokine coreceptor usage. J Infect Dis 179: 859–870. 10068581 10.1086/314660

[2] Shi M, Taylor JM, Fahey JL, Hoover DR, Munoz A, et al (1997) Early levels of CD4, neopterin, and beta 2-microglobulin indicate future disease progression. J Clin Immunol 17: 43–52. 9049785 10.1023/a:1027336428736

[3] Hazenberg MD, Otto SA, van Benthem BH, Roos MT, Coutinho RA, et al (2003) Persistent immune activation in HIV-1 infection is associated with progression to AIDS. Aids 17: 1881–1888. 12960820 10.1097/00002030-200309050-00006

[4] Goujard C, Bonarek M, Meyer L, Bonnet F, Chaix ML, et al (2006) CD4 cell count and HIV DNA level are independent predictors of disease progression after primary HIV type 1 infection in untreated patients. Clin Infect Dis 42: 709–715. 16447119 10.1086/500213

[5] Deeks SG, Kitchen CM, Liu L, Guo H, Gascon R, et al (2004) Immune activation set point during early HIV infection predicts subsequent CD4+ T-cell changes independent of viral load. Blood 104: 942–947. 15117761 10.1182/blood-2003-09-3333

[6] Stacey AR, Norris PJ, Qin L, Haygreen EA, Taylor E, et al.. (2009) Induction of a striking systemic cytokine cascade prior to peak viraemia in acute human immunodeficiency virus type 1 infection, in contrast to more modest and delayed responses in acute hepatitis B and C virus infections. J Virol: JVI.01844–01808. 10.1128/JVI.01844-08 PMC2663284 19176632

[7] Gay C, Dibben O, Anderson JA, Stacey A, Mayo AJ, et al (2011) Cross-Sectional Detection of Acute HIV Infection: Timing of Transmission, Inflammation and Antiretroviral Therapy. PLoS One 6: e19617. 21573003 10.1371/journal.pone.0019617 PMC3091862

[8] Kramer H, Lavender K, Qin L, Stacey A, Liu M, et al (2010) Elevation of intact and proteolytic fragments of acute phase proteins constitutes the earliest systemic antiviral response in HIV-1 infection. PLoS Pathog 6(5): e1000893. 20463814 10.1371/journal.ppat.1000893 PMC2865525

[9] Katsikis P, Mueller Y, Villinger F (2011) The Cytokine Network of Acute HIV Infection: A Promising Target for Vaccines and Therapy to Reduce Viral Set-Point? PLOS Pathog 7: e1002055–e1002055. 21852945 10.1371/journal.ppat.1002055 PMC3154847

[10] Kornfeld C, Ploquin MJ, Pandrea I, Faye A, Onanga R, et al (2005) Antiinflammatory profiles during primary SIV infection in African green monkeys are associated with protection against AIDS. J Clin Invest 115: 1082–1091. 15761496 10.1172/JCI23006 PMC1062895

[11] Ploquin MJ, Desoutter JF, Santos PR, Pandrea I, Diop OM, et al (2006) Distinct expression profiles of TGF-beta1 signaling mediators in pathogenic SIVmac and non-pathogenic SIVagm infections. Retrovirology 3: 37. 16800882 10.1186/1742-4690-3-37 PMC1533859

[12] Diop OM, Ploquin MJ, Mortara L, Faye A, Jacquelin B, et al (2008) Plasmacytoid dendritic cell dynamics and alpha interferon production during Simian immunodeficiency virus infection with a nonpathogenic outcome. J Virol 82: 5145–5152. 18385227 10.1128/JVI.02433-07 PMC2395206

[13] Meythaler M, Martinot A, Wang Z, Pryputniewicz S, Kasheta M, et al (2009) Differential CD4+ T-Lymphocyte Apoptosis and Bystander T-Cell Activation in Rhesus Macaques and Sooty Mangabeys during Acute Simian Immunodeficiency Virus Infection. J Virol 83: 572–583. 18987149 10.1128/JVI.01715-08 PMC2612394

[14] Campillo-Gimenez L, Laforge M, Fay M, Brussel A, Cumont MC, et al (2010) Nonpathogenesis of simian immunodeficiency virus infection is associated with reduced inflammation and recruitment of plasmacytoid dendritic cells to lymph nodes, not to lack of an interferon type I response, during the acute phase. J Virol 84: 1838–1846. 19939930 10.1128/JVI.01496-09 PMC2812402

[15] Jacquelin B, Mayau V, Targat B, Liovat AS, Kunkel D, et al (2009) Nonpathogenic SIV infection of African green monkeys induces a strong but rapidly controlled type I IFN response. J Clin Invest 119: 3544–3555. 19959873 10.1172/JCI40093 PMC2786805

[16] Favre D, Lederer S, Kanwar B, Ma ZM, Proll S, et al (2009) Critical loss of the balance between Th17 and T regulatory cell populations in pathogenic SIV infection. PLoS Pathog 5: e1000295. 19214220 10.1371/journal.ppat.1000295 PMC2635016

[17] Harris LD, Tabb B, Sodora DL, Paiardini M, Klatt NR, et al (2010) Downregulation of robust acute type I interferon responses distinguishes nonpathogenic simian immunodeficiency virus (SIV) infection of natural hosts from pathogenic SIV infection of rhesus macaques. J Virol 84: 7886–7891. 20484518 10.1128/JVI.02612-09 PMC2897601

[18] Durudas A, Milush JM, Chen HL, Engram JC, Silvestri G, et al (2009) Elevated levels of innate immune modulators in lymph nodes and blood are associated with more-rapid disease progression in simian immunodeficiency virus-infected monkeys. J Virol 83: 12229–12240. 19759147 10.1128/JVI.01311-09 PMC2786739

[19] Bosinger SE, Li Q, Gordon SN, Klatt NR, Duan L, et al (2009) Global genomic analysis reveals rapid control of a robust innate response in SIV-infected sooty mangabeys. J Clin Invest 119: 3556–3572. 19959874 10.1172/JCI40115 PMC2786806

[20] Estes JD, Li Q, Reynolds MR, Wietgrefe S, Duan L, et al (2006) Premature induction of an immunosuppressive regulatory T cell response during acute simian immunodeficiency virus infection. J Infect Dis 193: 703–712. 16453267 10.1086/500368

[21] Estes J, Gordon S, Zeng M, Chahroudi A, Dunham R, et al (2008) Early resolution of acute immune activation and induction of PD-1 in SIV-infected sooty mangabeys distinguishes nonpathogenic from pathogenic infection in rhesus macaques. J Immunol 180: 6798–6807. 18453600 10.4049/jimmunol.180.10.6798 PMC2596686

[22] Ananworanich J, Schuetz A, Vandergeeten C, Sereti I, de Souza M, et al (2012) Impact of multi-targeted antiretroviral treatment on gut T cell depletion and HIV reservoir seeding during acute HIV infection. PLOS one 7: e33948. 22479485 10.1371/journal.pone.0033948 PMC3316511

[23] Bebell L, Passmore J, Williamson C, Mlisana K, Iriogbe I, et al (2008) Relationship between levels of inflammatory cytokines in the genital tract and CD4+ cell counts in women with acute HIV-1 infection. J Infect Dis 198: 714–714. 10.1086/590503 18643751

[24] Roberts L, Passmore JA, Williamson C, Little F, Bebell LM, et al (2010) Plasma cytokine levels during acute HIV-1 infection predict HIV disease progression. Aids 24: 819–831. 20224308 10.1097/QAD.0b013e3283367836 PMC3001189

[25] Jiao Y, Zhang T, Wang R, Zhang H, Huang X, et al (2012) Plasma IP-10 Is Associated with Rapid Disease Progression in Early HIV-1 Infection. Viral Immunology 25: 333–337. 22788418 10.1089/vim.2012.0011

[26] Ghosn J, Deveau C, Chaix M, Goujard C, Galimand J, et al (2010) Despite being highly diverse, immunovirological status strongly correlates with clinical symptoms during primary HIV-1 infection: a cross-sectional study based on 674 patients enrolled in the ANRS CO 06 PRIMO cohort. Journal of Antimicrobial Chemotherapy 65: 741–748. 20167586 10.1093/jac/dkq035

[27] Troude P, Chaix M, Tran L, Deveau C, Seng R, et al (2009) No evidence of a change in HIV-1 virulence since 1996 in France; a study based on the CD4 cell count and HIV RNA/DNA levels at primary infection. AIDS 23: 1261–1267. 19424055 10.1097/QAD.0b013e32832b51ef

[28] Muñoz A, Sabin C, Phillips A (1997) The incubation period of AIDS. AIDS 11: S55–56. 9451969

[29] Nixon DE, Landay AL (2010) Biomarkers of immune dysfunction in HIV. Curr Opin HIV AIDS 5: 498–503. 20978393 10.1097/COH.0b013e32833ed6f4 PMC3032605

[30] Norris P, Pappalardo B, Custer B, Spotts G, Hecht F, et al (2006) Elevations in IL-10, TNF-alpha, and IFN-gamma from the earliest point of HIV Type 1 infection. AIDS Res & Hum Retroviruses 22: 757–762. 16910831 10.1089/aid.2006.22.757 PMC2431151

[31] Estes JD, Wietgrefe S, Schacker T, Southern P, Beilman G, et al (2007) Simian immunodeficiency virus-induced lymphatic tissue fibrosis is mediated by transforming growth factor beta 1-positive regulatory T cells and begins in early infection. J Infect Dis 195: 551–561. 17230415 10.1086/510852

[32] Herbeuval JP, Shearer GM (2007) HIV-1 immunopathogenesis: how good interferon turns bad. Clin Immunol 123: 121–128. 17112786 10.1016/j.clim.2006.09.016 PMC1930161

[33] Zeng M, Smith AJ, Wietgrefe SW, Southern PJ, Schacker TW, et al (2011) Cumulative mechanisms of lymphoid tissue fibrosis and T cell depletion in HIV-1 and SIV infections. J Clin Invest 121: 998–1008. 21393864 10.1172/JCI45157 PMC3049394

[34] Nakanishi K, Yoshimoto T, Tsutsui H, Okamura H (2001) Interleukin-18 regulates both Th1 and Th2 responses. Annu Rev Immunol 19: 423–474. 11244043 10.1146/annurev.immunol.19.1.423

[35] Mellors JW, Kingsley LA, Rinaldo CR Jr, Todd JA, Hoo BS, et al (1995) Quantitation of HIV-1 RNA in plasma predicts outcome after seroconversion. AnnIntMed 122: 573–579. 10.7326/0003-4819-122-8-199504150-00003 7887550

[36] Keating S, Golub E, Nowicki M, Young M, Anastos K, et al (2011) The effect of HIV infection and HAART on inflammatory biomarkers in a population-based cohort of women. AIDS 25: 1823–1832. 21572306 10.1097/QAD.0b013e3283489d1f PMC3314300

[37] Kamat A, Misra V, Cassol E, Ancuta P, Yan Z, et al (2012) A plasma biomarker signature of immune activation in HIV patients on anti-retroviral therapy. PLOS one 7: e30881. 22363505 10.1371/journal.pone.0030881 PMC3281899

[38] Theofilopoulos AN, Baccala R, Beutler B, Kono DH (2005) Type I interferons (alpha/beta) in immunity and autoimmunity. Annu Rev Immunol 23: 307–336. 15771573 10.1146/annurev.immunol.23.021704.115843

[39] Malleret B, Maneglier B, Karlsson I, Lebon P, Nascimbeni M, et al (2008) Primary infection with simian immunodeficiency virus: plasmacytoid dendritic cell homing to lymph nodes, type I interferon, and immune suppression. Blood 112: 4598–4608. 18787223 10.1182/blood-2008-06-162651

[40] Khatissian E, Chakrabarti L, Hurtrel B (1996) Cytokine patterns and viral load in lymph nodes during the early stages of SIV infection. Res Virol 147: 181–189. 8901438 10.1016/0923-2516(96)80233-0

[41] Thorburn E, Kolesar L, Brabcova E, Petrickova K, Petricek M, et al (2009) CXC and CC chemokines induced in human renal epithelial cells by inflammatory cytokines. APMIS 117: 477–487. 19594487 10.1111/j.1600-0463.2009.02446.x

[42] Foley JF, Yu CR, Solow R, Yacobucci M, Peden KW, et al (2005) Roles for CXC chemokine ligands 10 and 11 in recruiting CD4+ T cells to HIV-1-infected monocyte-derived macrophages, dendritic cells, and lymph nodes. J Immunol 174: 4892–4900. 15814716 10.4049/jimmunol.174.8.4892

[43] Piali L, Weber C, LaRosa G, Mackay CR, Springer TA, et al (1998) The chemokine receptor CXCR3 mediates rapid and shear-resistant adhesion-induction of effector T lymphocytes by the chemokines IP10 and Mig. Eur J Immunol 28: 961–972. 9541591 10.1002/(SICI)1521-4141(199803)28:03<961::AID-IMMU961>3.0.CO;2-4

[44] Cervantes-Barragán L, Firner S, Bechmann I, Waisman A, Lahl K, et al (2012) Regulatory T cells selectively preserve immune privilege of self-antigens during viral central nervous system infection. J Immunol 188: 3678–3685. 22407917 10.4049/jimmunol.1102422

[45] Lederman M, Margolis L (2008) The lymph node in HIV pathogenesis. Seminar Immunol 20: 187–195. 10.1016/j.smim.2008.06.001 PMC2577760 18620868

[46] Sarkar S, Kalia V, Murphey-Corb M, Montelaro R, Reinhart T (2003) Expression of IFN-gamma induced CXCR3 agonist chemokines and compartmentalization of CXCR3+ cells in the periphery and lymph nodes of rhesus macaques during simian immunodeficiency virus infection and acquired immunodeficiency syndrome. J Med Primatol 32: 247–264. 14498985 10.1034/j.1600-0684.2003.00031.x

[47] Lane B, King S, Bock P, Strieter R, Coffey M, et al (2003) The C-X-C chemokine IP-10 stimulates HIV-1 replication. Virology 307: 122–134. 12667820 10.1016/s0042-6822(02)00045-4

[48] Andersson J, Boasso A, Nilsson J, Zhang R, Shire NJ, et al (2005) The prevalence of regulatory T cells in lymphoid tissue is correlated with viral load in HIV-infected patients. J Immunol 174: 3143–3147. 15749840 10.4049/jimmunol.174.6.3143

[49] Mellors J, Margolick JB, Phair JP, Rinaldo CR, Detels R, et al (2007) Prognostic value of HIV-1 RNA, CD4 cell count, and CD4 Cell count slope for progression to AIDS and death in untreated HIV-1 infection. JAMA 297: 2349–2350. 17551128 10.1001/jama.297.21.2349

[50] Brenchley JM, Price DA, Schacker TW, Asher TE, Silvestri G, et al (2006) Microbial translocation is a cause of systemic immune activation in chronic HIV infection. Nat Med 12: 1365–1371. 17115046 10.1038/nm1511

[51] Lecuroux C, Girault I, Urrutia A, Doisne JM, Deveau C, et al (2009) Identification of a particular HIV-specific CD8+ T-cell subset with a CD27+ CD45RO−/RA+ phenotype and memory characteristics after initiation of HAART during acute primary HIV infection. Blood 113: 3209–3217. 19098272 10.1182/blood-2008-07-167601

[52] Benjamini Y, Hochberg Y (1995) Controlling the False Discovery Rate: a practical and powerful approach to multiple testing. J Royal Stat Soc Ser B 57: 289–300.

