# Supplementary figures and images for: Acute Plasma Biomarkers of T Cell Activation Set-Point Levels and of Disease Progression in HIV-1 Infection
Source: PLoS One. 2012 Oct 2;7(10):e46143. doi: 10.1371/journal.pone.0046143 (PMC3462744; doi:10.1371/journal.pone.0046143)

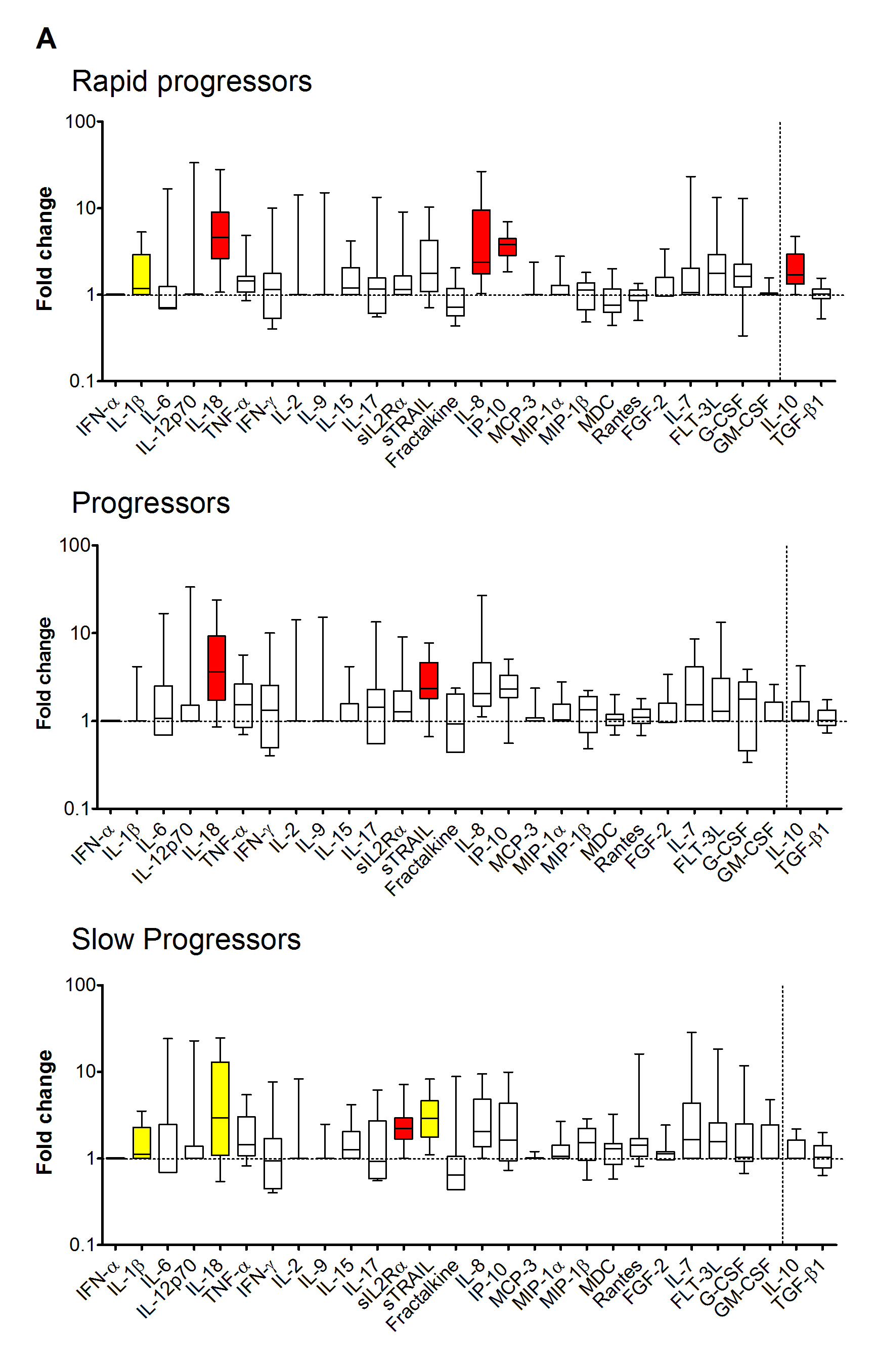


**
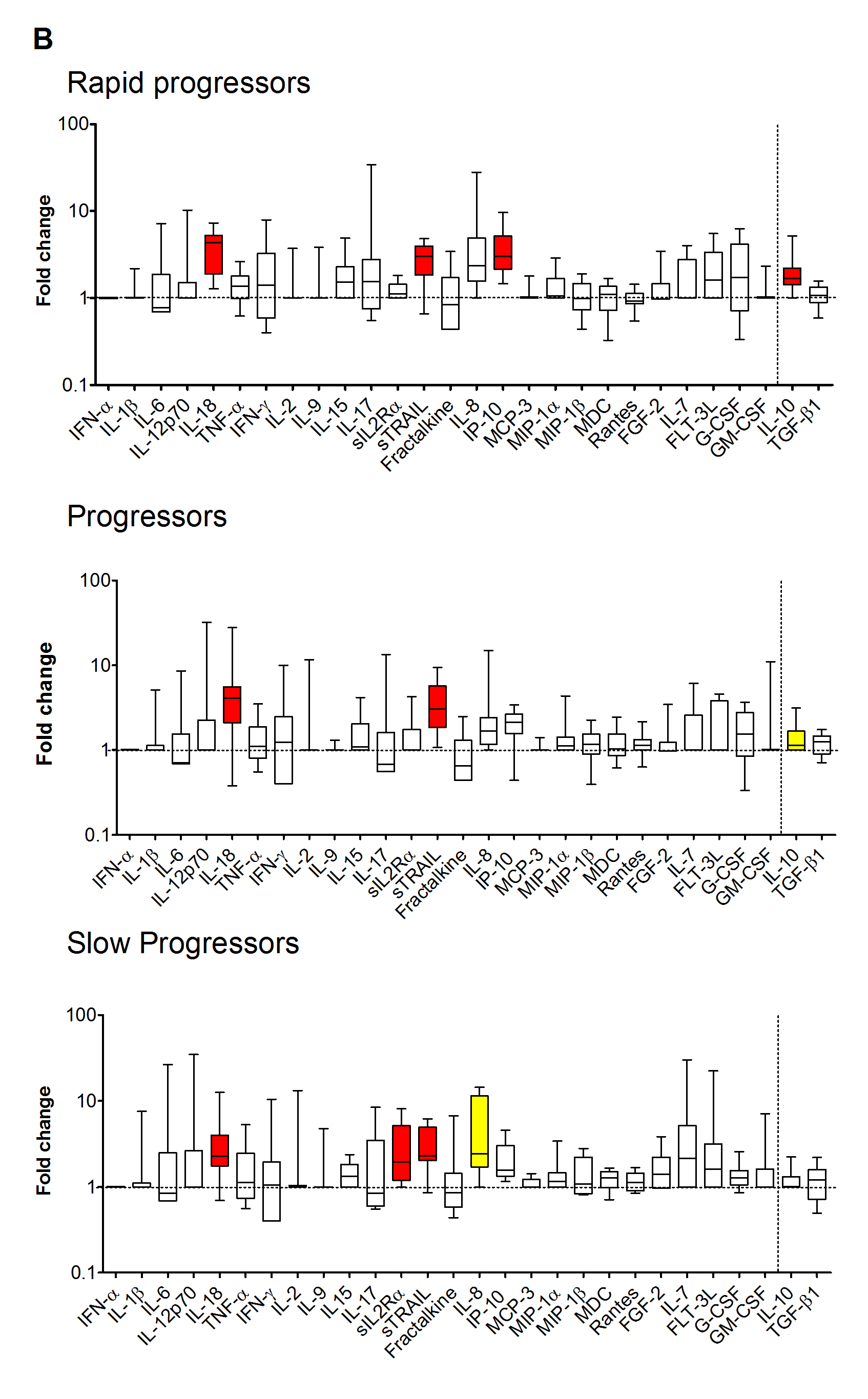
**

Supplement: Figure S1 — Plasma protein levels at M1 and M6 according to disease progression profiles. The plasma protein levels at M1 (A) and M6 (B) were expressed as fold change compared to those in healthy donors. Significant changes are indicated as red boxes (corrected threshold p<0.002). When a cytokine was increased (p<0.05), but the p-value was not under the corrected threshold (p<0.002) it was represented in a yellow box (p<0.005 on A and p<0.008 on B). The cytokines are listed from left to right according to their role (inflammatory, adaptive, IFN-inducible, chemoattractive, hematopoietic and anti-inflammatory). The dotted horizontal line corresponds to the respective values in healthy donors. (DOC) [file pone.0046143.s001.doc]

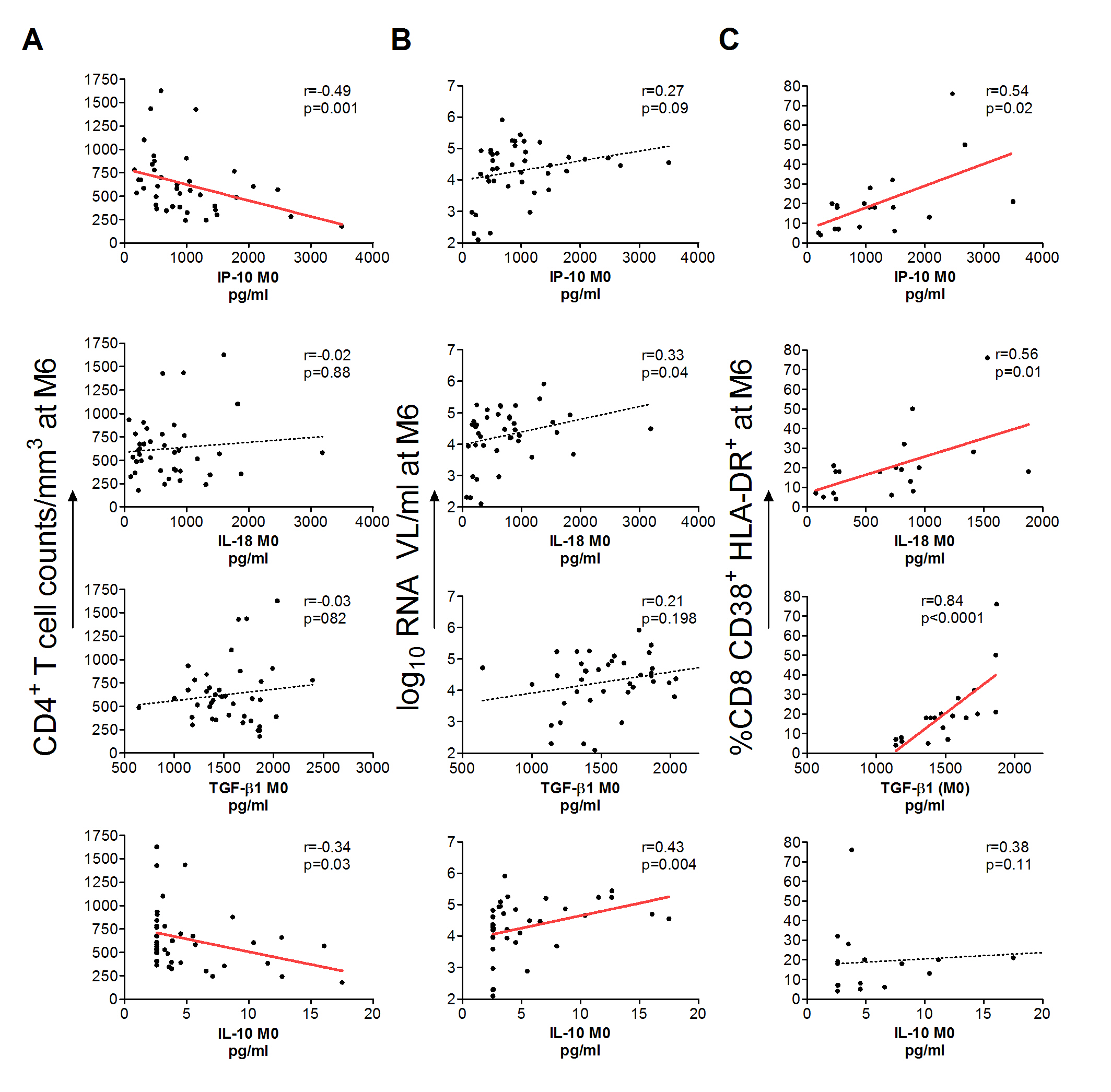

Supplement: Figure S2 — Correlation between plasma cytokine levels at M0 and disease progression markers. The cytokine concentrations at M0 have been plotted against (A) T CD4+ counts (N = 40), (B) viremia (N = 40) and (C) T cell activation (CD3+CD8+CD38+HLADR+, N = 19) levels at set point (M6). Here are represented the 5 cytokines (IP-10, IL-18, MCP-1, IL-10, TGF-β1) correlated with one (or more) of the disease progression markers (Spearman correlation). A red line indicates both a significant correlation and a significant linear regression. A black dotted line represents a non-significant linear regression. VL: viral load. (DOC) [file pone.0046143.s002.doc]

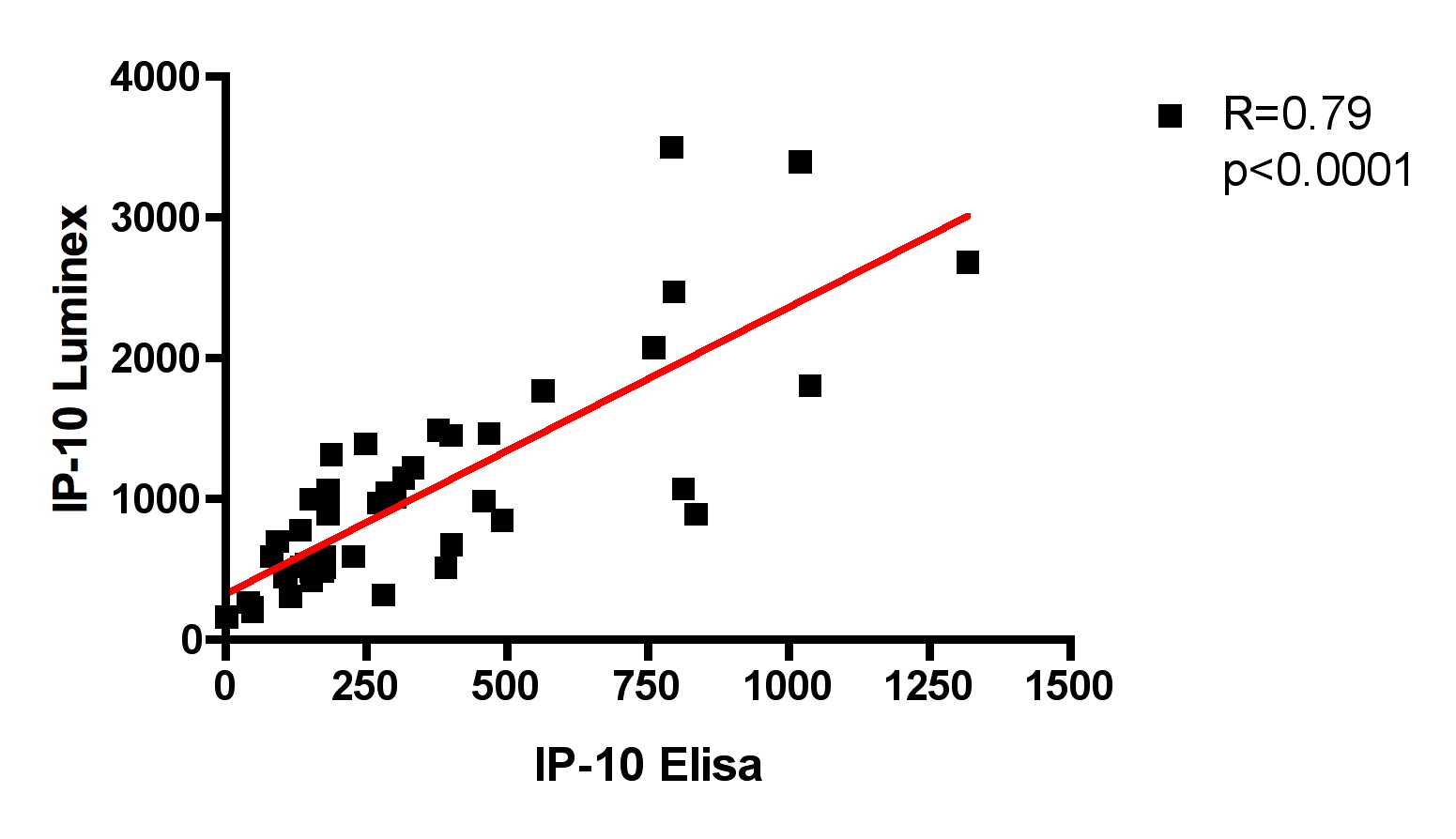

Supplement: Figure S3 — Correlation between IP-10 plasma concentrations quantified by Luminex and by Elisa. The IP-10 concentrations were determined in 45 patients during primary HIV-1 infection. (DOC) [file pone.0046143.s003.doc]
