# Supplementary material for: Acute Plasma Biomarkers of T Cell Activation Set-Point Levels and of Disease Progression in HIV-1 Infection
Source: PLoS One. 2012 Oct 2;7(10):e46143. doi: 10.1371/journal.pone.0046143 (PMC3462744; doi:10.1371/journal.pone.0046143)
Supplement: Table S1 — Demographic, clinical, virological and immunological characteristics of the 46 HIV-1 infected patients belonging to the derivation set. For each parameter, the median value is indicated. There were no significant differences between the groups concerning age (p = 0.65), gender (p = 0.32) or estimated time since infection (p = 0.4) (M&W test). In contrast, the three groups of patients presented differences in their T CD4+ counts and VL levels (see also Table S2). RP: Rapid progressors, P: progressors, SP: Slow progressors. The symbol § indicates a significant difference between SP and RP, * a difference between P and SP and # between RP and P (p<0.05). W: women, M: men, N: number of patients in each group. (DOC) [file pone.0046143.s004.doc]

**Table S1. Demographic, clinical, virological and immunological characteristics of the 46 HIV-1 infected patients belonging to the derivation set**

For each parameter, the median value is indicated. There were no significant differences between the groups concerning age (p=0.65), gender (p=0.32) or estimated time since infection (p=0.4) (M&W test). In contrast, the three groups of patients presented differences in their T CD4^+^ counts and VL levels (see also Table S2). RP: Rapid progressors, P: progressors, SP: Slow progressors. The symbol ^§^ indicates a significant difference between SP and RP, * a difference between P and SP and ^#^ between RP and P (p<0.05). W: women, M: men, N: number of patients in each group.
