# Supplementary material for: Acute Plasma Biomarkers of T Cell Activation Set-Point Levels and of Disease Progression in HIV-1 Infection
Source: PLoS One. 2012 Oct 2;7(10):e46143. doi: 10.1371/journal.pone.0046143 (PMC3462744; doi:10.1371/journal.pone.0046143)
Supplement: Table S2 — Comparison of viral load and T CD4+ counts between RP, P and SP of the derivation set. The VL and CD4+ T cell counts have been compared between the groups of patients described in Figure 1 and Table S1. The p-values are shown here for each comparison (M&W U-test). In red are indicated the statistically significant differences (p<0.05). M = month, RP = rapid progressor, P = progressor, SP = slow progressor. (DOC) [file pone.0046143.s005.doc]

**Table S2: Comparison of viral load and T CD4^+^ counts between RP, P and SP of the derivation set.**

|  | **SP/P** | | | | **SP/RP** | | | **P/RP** | | |
| --- | --- | --- | --- | --- | --- | --- | --- | --- | --- | --- |
|  | **M0** | **M1** | **M6** | **M42** | **M0** | **M1** | **M6** | **M0** | **M1** | **M6** |
| ***VL*** | 0.2 | 0.4 | 0.3 | 0.37 | **0.009** | **0.006** | **0.002** | 0.08 | **0.008** | **0.005** |
| ***CD4*** | **0.001** | **0.0003** | **0.0001** | **0.0028** | **0.00001** | **0.0002** | **0.0001** | **0.002** | 0.3 | **0.009** |

The VL and CD4^+^ T cell counts have been compared between the groups of patients described in Figure 1 and Table S1. The p-values are shown here for each comparison (M&W U-test). In red are indicated the statistically significant differences (p<0.05). M = month, RP = rapid progressor, P = progressor, SP = slow progressor.
