# Supplementary material for: Acute Plasma Biomarkers of T Cell Activation Set-Point Levels and of Disease Progression in HIV-1 Infection
Source: PLoS One. 2012 Oct 2;7(10):e46143. doi: 10.1371/journal.pone.0046143 (PMC3462744; doi:10.1371/journal.pone.0046143)
Supplement: Table S3 — Plasma protein profiles according to disease progression. The plasma proteins significantly elevated in one or more groups (RP for rapid progressors, P for progressors and SP for slow progressors) are shown here for the three time points: M0 (primary infection), M1 and M6. “+” stands for a significant difference as compared to healthy donors (M&W test, FDR corrected threshold: p<0.005 at M0, p<0.002 at M1 and M6). A “-“ stands for no change. M = month. (DOC) [file pone.0046143.s006.doc]

Table S3: Plasma protein profiles according to disease progression.

|  |  | **IL-1β** | **TNF-α** | **IL-8** | **IP-10** | **IL-10** | **IL-18** | **sTRAIL** | **sIL2Rα** |
| --- | --- | --- | --- | --- | --- | --- | --- | --- | --- |
| **M0** | **RP** | **+** | **+** | **+** | **+** | **+** | **+** | **+** | **+** |
|  | **P** | **-** | **-** | **-** | **+** | **+** | **+** | **+** | **-** |
|  | **SP** | **-** | **-** | **-** | **-** | **-** | **+** | **+** | **+** |
| **M1** | **RP** | **-** | **-** | **+** | **+** | **+** | **+** | **-** | **-** |
|  | **P** | **-** | **-** | **-** | **-** | **-** | **+** | **+** | **-** |
|  | **SP** | **-** | **-** | **-** | **-** | **-** | **-** | **-** | **+** |
| **M6** | **RP** | **-** | **-** | **-** | **+** | **+** | **+** | **+** | **-** |
|  | **P** | **-** | **-** | **-** | **-** | **-** | **+** | **+** | **-** |
|  | **SP** | **-** | **-** | **-** | **-** | **-** | **+** | **+** | **+** |

The plasma proteins significantly elevated in one or more groups (RP for rapid progressors, P for progressors and SP for slow progressors) are shown here for the three time points: M0 (primary infection), M1 and M6. “**+**” stands for a significant difference as compared to healthy donors (M&W test, FDR corrected threshold: p<0.005 at M0, p<0.002 at M1 and M6). A “-“ stands for no change. M = month.
