# Supplementary material for: Acute Plasma Biomarkers of T Cell Activation Set-Point Levels and of Disease Progression in HIV-1 Infection
Source: PLoS One. 2012 Oct 2;7(10):e46143. doi: 10.1371/journal.pone.0046143 (PMC3462744; doi:10.1371/journal.pone.0046143)
Supplement: Methods S1 — Luminex assay. Twenty-four plasma proteins were measured using a human cytokine Milliplex kit (Millipore): Fibroblast-growth-factor 2 (FGF-2), FMS-like-tyrosine-kinase-3-Ligand (FLT-3L), Fractalkine, Granulocyte-colony-stimulating factor (G-CSF), Granulocyte-macrophage-colony-stimulating factor (GM-CSF), Interferon γ (IFN-γ), Interleukin 1 β (IL-1β), IL-2, IL-6, IL-7, IL-8, IL-9, IL-10, IL-12p70, IL-15, IL-17, IP-10, macrophage-derived-chemokine (MDC), macrophage-inflammatory-protein 1α and β (MIP-1α, MIP-1β), monocyte-chemotactic-protein 3 (MCP-3), regulated-upon-activation-normal-T-cell-expressed and secreted (Rantes), soluble-IL2 receptor α (sIL2Rα) and tumor-necrosis-factor α (TNF-α). The limit of detection was 3.2 pg/ml except for: FLT-3L, G-CSF, GM-CSF, IL-7 and MIP-1β (16 pg/ml); FGF-2, MCP-3 and MIP-1α (40 pg/ml); and IP-10, Fractalkine and sIL2Rα (80 pg/ml). The standards (3.2 to 10,000 pg/ml) of the manufacturer were run on each plate in duplicate. All samples were assayed concurrently to avoid inter-assay variability. Data were acquired using a Luminex-100 system and analyzed using Bio-Plex Manager Software (Applied-Cytometry). (DOC) [file pone.0046143.s007.doc]

**Methods S1** : **Luminex assay**

Twenty-four plasma proteins were measured using a human cytokine Milliplex kit (Millipore): Fibroblast-growth-factor 2 (FGF-2), FMS-like-tyrosine-kinase-3-Ligand (FLT-3L), Fractalkine, Granulocyte-colony-stimulating factor (G-CSF), Granulocyte-macrophage-colony-stimulating factor (GM-CSF), Interferon γ (IFN-γ), Interleukin 1 β (IL-1β), IL-2, IL-6, IL-7, IL-8, IL-9, IL-10, IL-12p70, IL-15, IL-17, IP-10, macrophage-derived-chemokine (MDC), macrophage-inflammatory-protein 1α and β (MIP-1α, MIP-1β), monocyte-chemotactic-protein 3 (MCP-3), regulated-upon-activation-normal-T-cell-expressed and secreted (Rantes), soluble-IL2 receptor α (sIL2Rα) and tumor-necrosis-factor α (TNF-α). The limit of detection was 3.2 pg/ml except for: FLT-3L, G-CSF, GM-CSF, IL-7 and MIP-1β (16 pg/ml); FGF-2, MCP-3 and MIP-1α (40 pg/ml); and IP-10, Fractalkine and sIL2Rα (80 pg/ml). The standards (3.2 to 10,000 pg/ml) of the manufacturer were run on each plate in duplicate. All samples were assayed concurrently to avoid inter-assay variability. Data were acquired using a Luminex-100 system and analyzed using Bio-Plex Manager Software (Applied-Cytometry).
